# Metabolic Regulation of Redox Balance in Cancer

**DOI:** 10.3390/cancers11070955

**Published:** 2019-07-08

**Authors:** Vinee Purohit, Diane M. Simeone, Costas A. Lyssiotis

**Affiliations:** 1 Perlmutter Cancer Center, New York University, New York, NY 10016, USA; 2 Department of Surgery, New York University, New York, NY 10016, USA; 3 Department of Pathology, New York University, New York, NY 10016, USA; 4 Departments of Molecular and Integrative Physiology, University of Michigan, Ann Arbor, MI 48109, USA; 5 Internal Medicine, University of Michigan, Ann Arbor, MI 48109, USA; 6 Rogel Cancer Center, University of Michigan, Ann Arbor, MI 48109, USA

**Keywords:** oxidative stress, antioxidants, ROS, NADPH

## Abstract

Reactive oxygen species (ROS) are chemically active free radicals produced by partial reduction of oxygen that can activate discrete signaling pathways or disrupt redox homeostasis depending on their concentration. ROS interacts with biomolecules, including DNA, and can cause mutations that can transform normal cells into cancer cells. Furthermore, certain cancer-causing mutations trigger alterations in cellular metabolism that can increase ROS production, resulting in genomic instability, additional DNA mutations, and tumor evolution. To prevent excess ROS-mediated toxicity, cancer-causing mutations concurrently activate pathways that manage this oxidative burden. Hence, an understanding of the metabolic pathways that regulate ROS levels is imperative for devising therapies that target tumor cells. In this review, we summarize the dual role of metabolism as a generator and inhibitor of ROS in cancer and discuss current strategies to target the ROS axis.

## 1. Introduction

Oxygen (O_2_) is indispensable for the survival of eukaryotic organisms that use it to generate the energy currency adenosine triphosphate (ATP). O_2_ can also be lethal to cells through the generation of chemically reactive free radicals and ions, called reactive oxygen species (ROS). Recent studies have unfurled the role of ROS as signaling intermediates whose production and regulation are controlled processes with biological significance [1]. Discrepancies in the production or inhibition of ROS can cause defects in cell signaling, DNA damage, and lipid peroxidation. Hence, how a cell uses O_2_ while managing its potentially deleterious effects immediately impacts its physiology and survival. 

The ability of ROS to cause genomic instability makes it an inherent ally of cancer, and ROS induction is a well-established cause of carcinogenesis [2]. Indeed, early studies examining the functional role of ROS revealed that it has the capability to transform murine fibroblasts [3]. Transformation of cells to a malignant state causes a surge in ROS production, due to activation of oncogenes, loss of tumor suppressors, alterations in the expression, and/or assembly of mitochondrial electron transport chain (ETC) enzymes and through interaction with factors and conditions in the tumor microenvironment [4,5,6,7]. Increased ROS triggers further mutations in tumor cells, and promotes tumor growth, metastasis, and resistance to chemotherapies [8]. To survive and proliferate, cancer cells require mechanisms to balance this hyperactive ROS machinery. Hence, concomitant to increased ROS generation, cancer cells also increase the activity of antioxidant synthesis pathways, which enables them to survive in a microenvironment that is otherwise unfriendly to most normal adult cells. We will refer to this balance of ROS generation and ROS detoxification herein as ROS axis. 

Cellular metabolism governs various aspects of ROS axis and is an area of active exploration. In this review, we summarize recent work on the function of metabolism in the generation and detoxification of ROS in cancer. With the advent of novel therapies targeting altered metabolic pathways in cancer, a detailed understanding of ROS metabolism will provide valuable insights on how to harness this information to uniquely target neoplastic cells.

## 2. Cellular Metabolism as the Source of ROS

**Figure 1:**
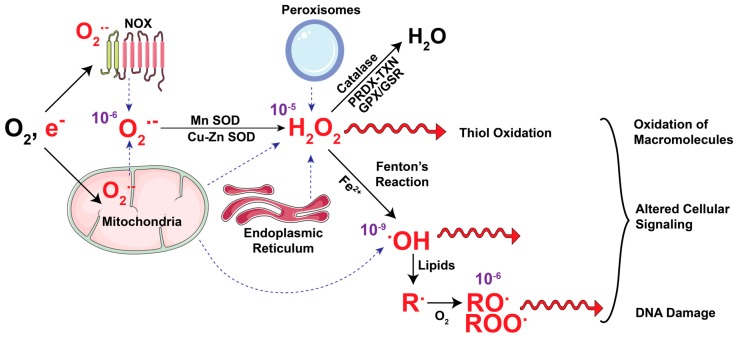
ROS axis: generation and detoxification of common reactive oxygen species. The mitochondrial electron transport chain (ETC) and NADPH oxidases (NOX) take up oxygen (O_2_) and generate superoxide (O_2_**^•−^**) which is dismutated in the mitochondria, peroxisome and endoplasmic reticulum by Copper-Zinc (Cu-Zn) and Manganese (Mn) superoxide dismutases (SOD) to generate hydrogen peroxide (H_2_O_2_). H_2_O_2_ is converted into water by simultaneous oxidation of the catalytic cysteine residues of the peroxiredoxin (PRDX) and thioredoxin (TXN) proteins. H_2_O_2_ is also actively nullified by the glutathione peroxidase-glutathione reductase (GPX-GSR) that recycles oxidized glutathione (GSSG, not shown) back to its reduced form (GSH, not shown). Mitochondrial O_2_**^•−^** radicals release Fe^+^ ions by damaging ETC complexes. In presence of Fe^2+^ ions H_2_O_2_ converts to hydroxyl radical (**^•^**OH) through Fenton’s reaction. Both H_2_O_2_ and **^•^**OH interacts and oxidizes macromolecules, alters cellular signaling and causes oxidative DNA damage, which drives gene mutations. **^•^**OH initiates oxidation of lipids to alkyl (R**^•^**) groups, which then oxidize to alkoxyl (RO**^•^**) and peroxyl (ROO**^•^**) radicals. (ROS is shown in red, dotted blue arrows indicate the source of ROS and purple numbers indicate half-life of these radicals in seconds).

ROS includes a wide array of free radical and non-radical oxygen containing species. Production of ROS in cells encompasses one-step reduction of molecular oxygen to generate superoxide ion (O_2_**^•−^**) (Figure 1). O_2_**^•−^** then undergoes a multistep detoxification process generating other ROS molecules, which include hydroxyl radical (**^•^**OH), nitric oxide (NO**^−^**), alkoxyl (RO**^•^**), peroxyl (ROO**^•^**), singlet oxygen (^1^O_2_), and non-radical hydrogen peroxide (H_2_O_2_). The effect of ROS in cells is contingent upon their cellular location, concentration, and half-life. O_2_**^•−^** is generated first and rapidly dismutates, especially at low pH to H_2_O_2,_ the primary mediator of ROS-driven cellular signaling. The longer half-life of H_2_O_2_ enables it to diffuse through aquaporins in the cell membranes and oxidize cysteine residues in proteins, a post-translational modification that can change their structure and functions. In the presence of Fe^2+^ ions, H_2_O_2_ undergoes Fenton’s reaction generating highly reactive **^•^**OH that can cause rapid oxidative damage of lipids, DNA, and proteins. 

Cellular enzymes, including those that catalyze mitochondrial oxidative metabolism, NADPH oxidases (NOX), nitric oxide synthases, cyclooxygenases, xanthine oxidase, lipoxygenases and cytochrome p450, are important mediators and sites of ROS production. Among organelles, mitochondria, peroxisomes, and the endoplasmic reticulum are the primary locations of ROS production in cells. In the section that follows, we detail the three predominant sources of cellular ROS in both these groups, i.e., enzymes and organelles, with a focus on metabolic pathways that mediate ROS generation.

### 2.1. Mitochondrial ROS

**Figure 2:**
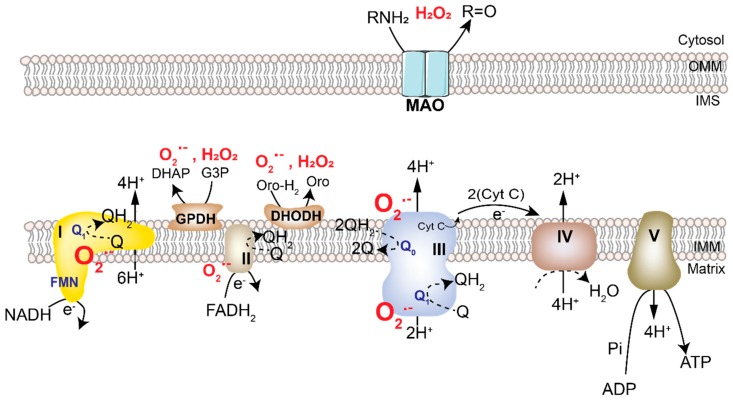
Mitochondrial sites that generate ROS. Mitochondria generate cellular energy in the form of ATP via the transfer of electrons through the electron transport chain (ETC), which is a set of multi-protein assemblies known as complexes I–IV. These exist in inner mitochondrial membrane (IMM). NADH and FADH_2_ generated in the tricarboxylic acid (TCA) cycle enter the ETC at complex I and II, respectively, and generate protons and electrons during their conversion to NAD+ and FAD. The protons generated in the process are released into the intermembrane space (IMS), where a proton gradient is established. These electrons are taken up by FMN or FAD in complex I and II, respectively, and are transferred to the iron-sulfur clusters finally generating quinol (QH_2_) from quinone (Q). In complex IV (Cytochrome C-oxidase), the electrons are transferred to oxygen generating water molecules. The electrochemical energy stored in the proton gradient is used to drive ATP synthase (also called complex V), which simultaneously converts matrix ADP to ATP by addition of inorganic phosphate (Pi). At complexes I, II and III of the ETC, a small amount of oxygen undergoes one-step reduction to generate superoxide ion (O_2_^•−^), which is released in the matrix or IMS. Other mitochondrial enzymes generate O_2_**^•−^**or dismutate it to generate H_2_O_2_. These include Glycerol-3-phosphate dehydrogenase (GPDH) and dihydroorotate dehydrogenase (DHODH) on the IMM, and monoamine oxidases (MAO) in the outer mitochondrial membrane (OMM). (ROS is shown in red).

Mitochondria are an important source of cellular ROS that contributes both to the physiological redox signaling from this organelle as well as oncogenesis. The primary function of mitochondria is to oxidize substrates in the tricarboxylic acid (TCA) cycle along with the concomitant reduction of flavin adenine dinucleotide (FADH_2_) and nicotinamide adenine dinucleotide (NADH). The electrons generated are then transferred through the multiprotein complexes of the ETC driving an electrochemical proton gradient (Figure 2). The energy stored in this gradient is harnessed either by ATP synthase (Complex V) to generate ATP or uncoupling proteins (UCP) to generate heat. However, electrons frequently leak out of the ETC, converting up to 1.0% (0.1–1%) of molecular oxygen into O_2_**^•−^** [9,10]. O_2_**^•–^** is subsequently converted into H_2_O_2_ and **^•^**OH. As the ETC and several ROS-generating enzymes are localized to the mitochondria, they are one of the most consistent and significant producers of cellular ROS [11,12]. To maintain ROS generation and a functional ETC, mitochondria have antioxidant enzymes and reducing equivalents discussed later in the review. 

The O_2_**^•−^** ion exhibits a high electrostatic attraction toward Fe-S clusters, which are integral components of mitochondrial complexes. Interaction with O_2_**^•−^** degrades Fe-S clusters and impairs proper mitochondrial respiration. Mitochondrial, as well as cytoplasmic, H_2_O_2_ plays an important signaling role via oxidation of cysteine residues in several proteins, including transcription factors and phosphatases. Like O_2_**^•−^**, H_2_O_2_ also disrupts mitochondrial iron metabolism and increases intracellular levels of labile iron [13]. This increased labile iron drives Fenton’s reaction to generate **^•^**OH which disrupts macromolecules including lipids and DNA, the latter of which drive further mutations to promote cancer growth (Figure 1) [13]. 

ETC complexes I and III produce the majority of mitochondrial ROS of which only complex III releases O_2_**^•−^** to both the matrix and intermembrane space (Figure 2) [9]. Weinberg et al. demonstrated that the Q_0_ site of mitochondrial complex III is the major source of cellular ROS and is essential for oncogenic KRAS-mediated anchorage-independent growth and tumorigenesis [5]. Within complex III, transfer of electrons from the Q_0_ site to the Rieske-Fe-S-Proteins (RISP) generates mitochondrial ROS. Cells lacking RISP fail to perform oxidative phosphorylation (OXPHOS) as well as to generate ROS, unlike those lacking cytochrome b, which only have defect in oxygen consumption [9]. In Lewis lung carcinoma cells, mutation in the mitochondrial DNA encoded gene for complex I leads to ROS induction, which is required for tumor metastasis [14]. Not surprisingly, treatment with mitochondrial ETC inhibitors also increases cellular ROS and leads to cell death in some circumstances. However, the mechanisms mediating cell death under these conditions could be independent of ROS and rather result from direct ETC inhibition [15]. 

In addition to the ETC, other mitochondrial enzymes can also generate ROS in the form of O_2_**^•−^** or H_2_O_2_ [16]. These include dihydroorotate dehydrogenase (DHODH) and glycerol-3-phosphate dehydrogenase (GPDH) on the inner mitochondrial membrane, and monoamine oxidases (MAO) on the outer mitochondrial membrane (Figure 2). DHODH, an enzyme involved in the de novo synthesis of pyrimidines, is located in the mitochondria, functions in close coordination with complex III, and can generate O_2_^•−^ or H_2_O_2_. In preclinical models, inhibition of DHODH has been shown to increase the efficacy of chemotherapy by significantly enhancing cell death in pancreatic tumors refractory to therapies or ovarian, lung, pancreatic, and colon cancer cells with oncogenic Kras [17,18]. GPDH is an important ROS producing mitochondrial flavin dehydrogenase. ROS production by this enzyme has been attributed in part to glycerol-3-phosphate-dependent reverse electron transport [19]. Although the mechanism remains incompletely understood, targeting GPDH has been reported to inhibit proliferation in prostate cancer cells [20]. Mitochondrial MAO-mediated oxidative deamination of catecholamines, serotonins, and dietary amines generates H_2_O_2_ [21]_._ Upregulation of these enzymes has been linked with increased tumorigenesis and metastasis in prostate cancer [22]. While strategies to target these enzymes have been proposed, further studies are warranted to understand the precise biology of ROS production and implications of targeting MAOs in biological systems. 

### 2.2. NADPH Oxidases

NADPH oxidases (NOXs) are membrane-associated flavocytochrome proteins and the largest single producers of ROS in some cell types. The physiological function of NOX to directly produce ROS was first identified in phagocytes [23]. NOXs facilitate the transfer of an electron from NADPH to the FAD cofactor, from which the electron is then transferred to a heme group and lastly to oxygen to generate O_2_**^•−^**. NADPH binds at the cytoplasmic N-terminus of NOX whereas ROS is generated in either the extracellular or intra-organelle space. Most NOX enzymes produce ROS intracellularly except NOX1, which produces ROS in the extracellular space and through a poorly understood mechanism activates endocytosis to re-import ROS into the cytoplasm [24]. This enables the cell to direct ROS production, and in the case of phagocytes, to target ROS against pathogens. 

The NOX complex is a multisubunit complex. Depending on the tissue type, the catalytic subunits exist as NOX1-5 and dual membrane oxidases (DUOX 1 and 2), the latter being specific to the thyroid [23]. 

Several instances have been reported in which cancer cells have hijacked the NOXs to drive ROS formation to support tumorigenesis. For example, mutant KRAS activity leads to increased NOX1 expression, and the NOX-dependent generation of ROS is essential for KRAS-induced cellular transformation in fibroblasts [25,26]. A study by Park et al. demonstrated that the mutant Kras dependent phosphorylation of P47^phox^, the regulatory subunit of NOX, on Ser^348^ and Ser^379^ facilitated its localization to cell membrane to activate NOX1-mediated intracellular ROS production and malignant transformation. Similar studies have revealed that expression of NOX1 is increased in colon adenocarcinoma and correlates with KRAS mutation status [27]. Like KRAS in colon cancers, HRAS increases intracellular O_2_^•−^ levels by activating membrane-associated NOXs in human lung cells [28]. Hypoxia-inducible factor 1-alpha (HIF1α) also directly regulates the expression of *NOX1* and increases the expression of NOX2 [29,30]. Hypoxia and other oncogenic signaling pathways activate HIF1α in many solid tumors. Hence activation of NOX1 and NOX2 may be a general feature of many cancer types. Moreover, ROS generated by NOX can potentiate HIF1α by inhibiting its proteasomal degradation. Like other NOX enzymes, NOX4 also plays a pro-cancer role, and it is up-regulation has been reported in renal cell carcinoma [31], melanoma [32], glioblastoma multiforme (GBM) [33], ovarian [34], and pancreatic cancer [35]. Similarly, ROS signaling by NOX5 increases proliferation and survival of prostate cancer [36]. In addition, increased DUOX expression and signaling has been reported in thyroid [37] and pancreatic ductal adenocarcinomas [38].

### 2.3. Peroxisomal Metabolism and Oxidative Stress

Peroxisomes function as intracellular vesicles containing H_2_O_2_, a feature from which their name is derived. However, the ambit of peroxisomal functions extends far beyond H_2_O_2_ retention. Peroxisomal xanthine oxidase is an enzymatic source of H_2_O_2_ and O_2_^•−^. Additionally, peroxisomes have other H_2_O_2_-producing enzymes, including polyamine oxidases, aspartate oxidases, and hydroxyl acid oxidases. Peroxisomes frequently occur in the vicinity of lipid droplets in the cells and regulate cellular lipid metabolism, favoring the β-oxidation of fatty acids (fatty acid oxidation, FAO). These organelles contain enzymes involved in long chain fatty acid (LCFA) oxidation, amino acid oxidation and purine catabolism [39]. Peroxisomes take up very long chain fatty acid (VLCFA) and break these down to LCFA using the cofactor FAD. Unlike the mitochondrial ETC, the FAD in peroxisomes can directly reduce molecular oxygen to H_2_O_2_, making this an equivalent to mitochondrial OXPHOS−ETC, but lacking ATP generation. 

Proliferating cells require external and ER-generated unsaturated lipids [40], the former being provided by adipocytes or fibroblasts [41,42]. To diminish the loss of lipids by FAO in peroxisomes, cancer cells regulate peroxisome function and ROS through the peroxisome specific autophagy program, known as pexophagy. A recent study in VHL-deficient renal cell carcinomas demonstrated that HIF-2α stabilization in these cancers increased localization of autophagy receptor Nbr1 on peroxisomes, increasing pexophagy [43]. Consistently, Vhl^−/−^ mice have increased hepatic VLCFA and very long chain poly-unsaturated fatty acids (VLC-PUFA), reflecting the absence of peroxisomes due to pexophagy [43]. Additionally, HIF-2α also deactivates PPARα-mediated peroxisome biogenesis. PPAR agonists have the potential to increase peroxisome numbers and ROS, mediating increase in saturated fatty acid, protein/nucleotide catabolism, ROS, and cancer cell death. However, despite the preclinical efficacy of PPAR agonists, the recent phase II clinical trial of fenofibrate in smoldering multiple myeloma patients yielded no clinical benefits (NCT01965834). Considering these results, the clinically ready HIF-2α inhibitors could provide an additional means to inhibit peroxisomal ROS in cancers. 

## 3. Metabolic Pathways That Mitigate ROS

Aberrant ROS production can result in oxidative imbalance that is sufficient to harm or kill cancer cells. To prevent this from occurring, cancer cells activate cellular antioxidant pathways. One major mechanism by which this occurs is through the activation of the transcription factor and master regulator of antioxidant response, Nuclear factor erythroid 2-related factor 2 (NRF2). Under redox homeostasis, NRF2 binds to Kelch-like ECH-associated protein1 (KEAP1), is ubiquitinated and targeted for degradation [44,45]. To overcome this, cancer cells hyperactivate NRF2 signaling by increasing NRF2 mRNA expression [46] and/or impairing KEAP1-NRF2 interaction, which stabilizes NRF2 protein. NRF2-KEAP1 interaction is inhibited by (1) somatic mutations in KEAP1, Cullin3 (CUL3), or NRF2 genes, including 10%–15% lung [47,48], and 8% hepatocellular [49] cancers, (2) epigenetic silencing of *KEAP1* [50], (3) accumulation of KEAP1 or NRF2 interacting proteins, such as BRCA1, p62 or PALB2 [51,52,53], (4) cysteine modification of KEAP1 mediated by oncometabolites, such as fumarate [54] and itaconate [55], or (5) modification of KEAP1 by glycolysis-derived methylglyoxal [56]. Cancer cells employ one or more of these mechanisms to regulate NRF2 activity and maintain redox flux. 

**Figure 3:**
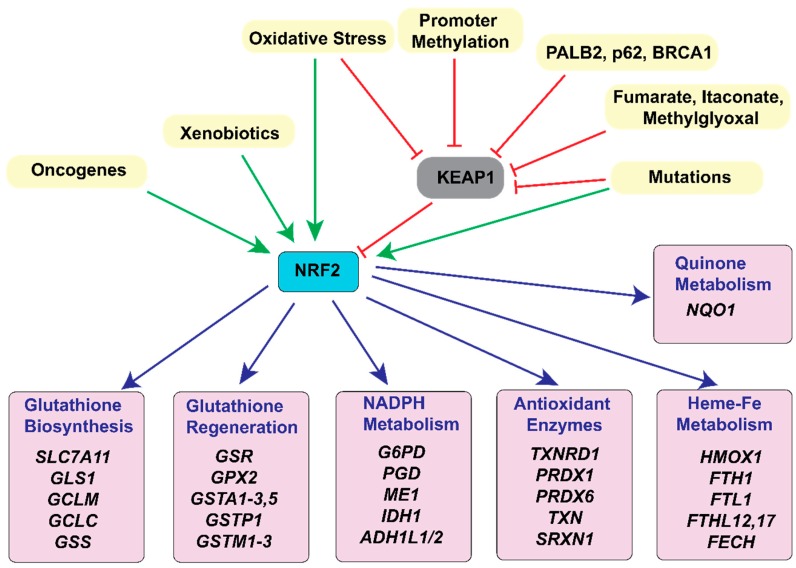
KEAP1-NRF2 in the regulation of metabolism and cellular antioxidant response. Nuclear factor erythroid 2-related factor 2 (NRF2) is the regulator of antioxidant response in cancer. NRF2 is activated by gene mutations, oncogene activation, xenobiotics, or oxidative stress. Oxidative stress also inactivates the principal NRF2 inhibitor in cells, Kelch-Like ECH-associated protein 1 (KEAP1), by cysteine oxidation. KEAP1 is also inhibited by mutations, promoter methylation, binding with KEAP1-sequestering proteins like PALB2, BRCA1, and p62, and interaction with metabolites such as fumarate, itaconate, and methylglyoxal. Stabilized NRF2 regulates expression of several genes involved in redox homeostasis. Shown here are NRF2-target genes involved directly in redox balance. First are genes involved in glutathione (GSH) biosynthesis: solute carrier family 7 member 11 (SLC7A11), glutaminase1 (GLS1), glutamate-cysteine ligase, modifier subunit (GCLM), glutamate-cysteine ligase catalytic subunit (GCLC), and glutathione synthetase (GSS). Glutamate generated by GLS1, a NRF2 target gene, can also be utilized for GSH biosynthesis. The second set has genes involved in GSH recycling: glutathione-disulfide reductase (GSR), glutathione peroxidase 2 (GPX2), glutathione S-transferase Alpha (GSTA), glutathione S-transferase Pi (GSTP), and glutathione S-transferase Mu (GSTM). Third set has enzymes mediating NADPH metabolism: glucose-6-phosphate dehydrogenase (G6PD), phosphogluconate dehydrogenase (PGD), malic enzyme (ME1), isocitrate dehydrogenase (IDH1), aldehyde dehydrogenase 1 family, and member L1 and L2 (ADH1L1/2). Other metabolic enzymes shown in the 4th set are: thioredoxin 1 (TXN1), thioredoxin reductase 1 (TXNRD1), peroxiredoxin 1 (PRDX1), peroxiredoxin 6 (PRDX6) and sulfiredoxin 1 (SRXN1). Fifth/Sixth set are enzymes in Heme, Iron (Fe) and quinone metabolism: heme oxygenase 1 (HMOX1), ferritin heavy chain 1 (FTH1), ferritin light chain 1 (FTL1), ferritin heavy chain 1 pseudogene 12 (FTHL12), ferritin heavy chain 1 pseudogene 17 (FTHL17), ferrochelatase (FECH) and NAD(P)H quinone dehydrogenase 1 (NQO1). (Green arrows indicate direct and red arrows indirect mechanisms of NRF2 activation. Blue arrows direct to genes transcriptionally regulated by NRF2).

The ROS-mediated NRF2 response activates each of the principal classes of antioxidants (Figure 3): NADPH, glutathione (GSH-GSSG), and the peroxiredoxin-thioredoxin (PRDX-TXN) and other such antioxidant enzyme systems [57,58]. In the section that follows, we provide an overview of these three antioxidant classes, present the metabolic pathways that lead to their function and regulation, including but not limited to NRF2, and discuss how these pathways are differentially regulated in cancer cells.

### 3.1. NADPH

Enzyme-catalyzed redox reactions are assisted by biomolecules known as coenzymes. The coenzyme nicotinamide adenine dinucleotide phosphate (NADPH) is a major bioenergetic and redox equivalent in cells, which can be generated by the following five metabolic pathways. Another such coenzyme regulating redox in cancers is NADH, for a detailed understanding of which, the reader is referred to a recent detailed review [59]. Unlike NADH, NADPH is compartmentalized in the cells with no reported transport or exchange between mitochondrial and cytosolic pools [60].

**Figure 4:**
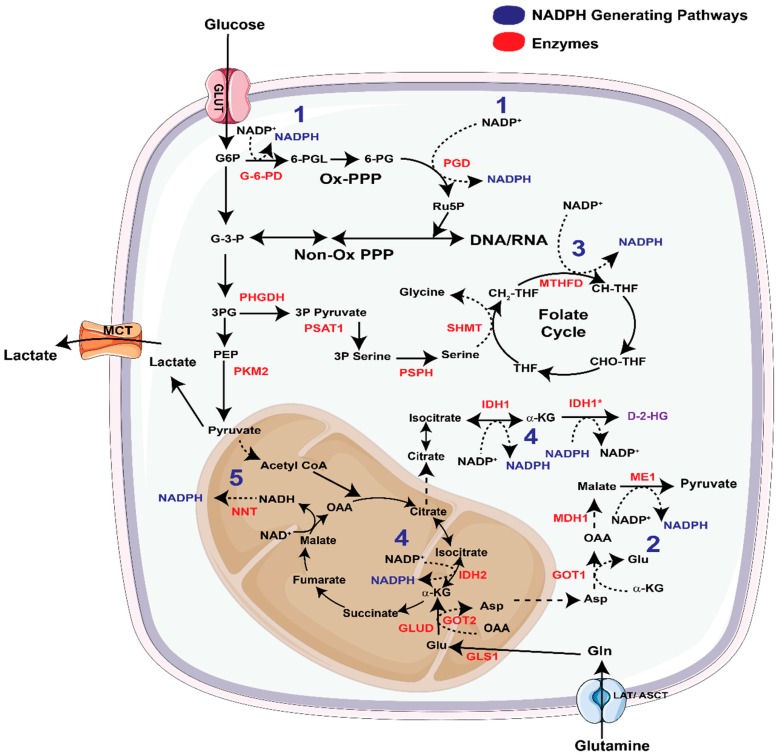
Altered metabolism as the source of nadph biosynthesis. The principal metabolic pathways that generate NADPH are numbered in blue with key enzymes shown in red. These include (1) the pentose phosphate pathway (PPP) in which conversion of Glucose-6-phosphate (G6P) to 6-phosphogluconolactone (6-PGL) and 6-phosphogluconate (6-PG) to ribulose-phosphate (Ru5P) generates 2 NADPH molecules. (2) Another mechanism of NADPH production is via the conversion of malate to pyruvate by malic enzyme 1. (3) The third reaction mediating NADPH production is the methylenetetrahydrofolate dehydrogenase (MTHFD)-mediated conversion of 5,10-methylenetetrahydrofolate (CH_2_-THF) to methenyltetrahydrofolate (CH-THF). (ME1). (4) Iso-citrate dehydrogenases (IDH) and (5) nicotinamide nucleotide transhydrogenases (NNT) are other routes of NADPH production in cancer cells. (LAT = Large neutral amino acid transporter, ASCT = Alanine, Serine Cysteine transporter, MCT = monocarboxylate transporters, GLUT = glucose transporter, G-3-P = glyceraldehyde-3-phosphate, 3P serine = 3 phosphoserine, 3P pyruvate = 3 phosphopyruvate, 3PG = 3 phosphoglycerate, PEP = Phosphoenolpyruvate, THF = tetrahydrofolate, CHOTHF = 10-formyl-tetrahydrofolate, OAA = oxaloacetate, Gln = glutamine, Glu = glutamate, α-KG = α-Ketoglutarate, Asp= Aspartic acid, G-6-PD = glucose-6-phosphate dehydrogenase, PGD = phosphogluconate dehydrogenase, PHGDH = phosphoglycerate dehydrogenase, PSAT1= phosphoserine aminotransferase 1, PSPH = phosphoserine phosphatase, SHMT = serine hydroxymethyltransferase, GLS = glutaminase, GLUD = glutamate dehydrogenase, GOT1/2 = glutamic oxaloacetic transaminase, MDH1 = malate dehydrogenase 1, IDH1* = mutant isocitrate dehydrogenase, D-2-HG = D-2-hydroxyglutarate).

*The Pentose Phosphate Pathway:* The pentose phosphate pathway (PPP) has two branches, i.e., the oxidative and the non-oxidative branch (Figure 4) of which the former generates NADPH. Oncogenes frequently modulate the flux of metabolites through one of these two branches to cater to the metabolic needs of cancer cells. For example, in pancreatic cancer cells, oncogenic KRAS increases the flux of glucose carbon through the non-oxidative PPP to favor nucleotide biosynthesis, without altering flux of glucose carbon to the oxidative PPP (ox-PPP). Increase in non-oxidative PPP activity supports the growth of KRAS-transformed pancreatic tumors [61]. However, recent studies have elegantly shown that oncogenic KRAS-directed flux of glucose carbon into PPP depends on the spatial location of tumors. For example, glucose utilization for PPP flux in pancreatic cancer varies significantly in the distant metastatic sub-clones in comparison to the primary and locally metastasized tumors [62]. Using patient-derived matched primary and metastatic samples, McDonald and Li et al. elucidated that unlike primary and peritoneal clones, distant metastases, and their precursor sub-clones prefer utilizing the oxidative branch of the PPP. Therefore, targeting 6-phosphogluconate dehydrogenase (PGD), one of the two NADPH producing enzymes in the ox-PPP, presents a therapeutic strategy in metastatic pancreatic cancer. 

The tumor suppressor p53 (TP53) both suppresses and activates the ox-PPP. Wild-type (WT) p53 protein interacts with and inhibits the formation of the dimeric glucose-6-phosphate dehydrogenase (G6PD) holoenzyme, thereby inhibiting the ox-PPP in unstressed human colorectal cancer cells [63]. WT TP53-induced glycolysis and apoptosis regulator (TIGAR) lowers the levels of fructose-2,6-bisphosphate (FBP), an allosteric activator of phosphofructokinase 1, and inhibits glycolysis redirecting the glucose-derived carbons through the ox-PPP [64]. P53 also binds with phosphoglycerate mutase and inhibits ox-PPP, a property absent in dominant negative p53 mutant cells [64,65]. These studies illustrate that the role of p53 in regulating the PPP are context and mutation dependent. Tuberous sclerosis proteins 1 and 2 (TSC1/2), which leads to the activation of the mechanistic target of rapamycin (mTOR) signaling, also drives ox-PPP-mediated NADPH synthesis, although in a Sterol regulatory element-binding protein (SREBP)-mediated fashion [66].

*Malic Enzymes:* Oncogenic KRAS promotes the predilection to non-oxidative PPP in local and locally metastasized tumors, thereby bypassing the NADPH-generating oxidative PPP in these cases [61]. Accordingly, mutant KRAS-expressing pancreatic cancers utilize and rely on an alternative pathway to synthesize NADPH. In pancreatic cancer, unlike in other cancers [67], glutamate is converted into alpha ketoglutarate (α-KG) by mitochondrial aspartate transaminase (GOT2), as opposed to glutamate dehydrogenase (GLUD1) (Figure 4). This reaction concomitantly produces aspartate (Asp), which is subsequently shuttled to the cytosol. Here, cytosolic aspartate transaminase or glutamic oxaloacetic transaminase 1(GOT1) converts Asp into oxaloacetate (OAA) and cytosolic malate dehydrogenase (MDH1) converts this OAA into malate. The malate is finally converted into pyruvate and NADPH by cytosolic malic enzyme (ME1) (Figure 4). KRAS-induced pancreatic cancers depend on the ME1-mediated NADPH generation to maintain redox balance and proliferation. Inhibition of this pathway significantly diminishes the pancreatic tumor growth and can be rescued by antioxidant supplementation [68].

The tumor suppressor protein p53 has been shown to repress the expression of ME1 and ME2, the latter being the mitochondrial isoform of the malic enzyme. Jiang et al. revealed that *TP53*-knockdown increases cellular NADPH levels, which are diminished upon inhibition of ME1 and even more dramatically for ME2 [69]. ME1 and ME2 were also found to regulate glutamine metabolism in P53^−/−^ HCT116 cells [69]. By generating knockdowns of ME1 or ME2, the authors observed that both ME1 and ME2 regulate glucose consumption and glutaminolysis in these cells. However, glutamine uptake was preferentially regulated by ME2. In another study, Ren et al. reported that targeting ME2 increases ROS and the NADP+/NADPH ratio, inducing cell death in A549 lung cancer cells [70].

In lung and breast cancers glutamate dehydrogenase 1 (GLUD1), instead of GOT1/2, produces α-KG and subsequently fumarate, which activates glutathione peroxidase 1, regulating the cellular antioxidant response at an additional level [71]. 

*Methylene tetrahydrofolate dehydrogenase (MTHFD)*: The serine biosynthetic pathway generates the amino acids serine and glycine from the glycolytic intermediate 3-phosphoglycerate (3-PG). Serine and glycine are important metabolites in the 1-carbon pathway, which provides methylation intermediates for epigenetic regulation and de novo nucleotide biosynthesis. Indeed, the serine biosynthesis pathway is activated in a number of cancers, and its presumptive function is to fuel the 1-carbon pathway and nucleotide biosynthesis [72,73,74,75,76,77,78]. It is now well established that this pathway is also an important source of NADPH especially in certain cancer contexts [79]. The generation of NADPH in this pathway is initiated by the serine hydroxymethyltransferase (SHMT). Both cytosolic SHMT1 and mitochondrial SHMT2 (both shown as SHMT in Figure 4) transfer methyl groups from serine to tetrahydrofolate (THF) to generate glycine and 5,10-methylene THF. The 5,10-methylene THF is converted into methylene THF by methylene THF dehydrogenase (MTHFD1 in the cytosol and MTHFD2 and MTHFD2L in the mitochondria) thereby producing NAD(P)H (Figure 4). Conversion of serine to glycine and THF, by SHMT2 and MTHFD2 respectively contribute to glycine and NADPH pools in mitochondria [80]. Inhibition of mitochondrial MTHFD2/2L makes cancer cells dependent on external serine and cytoplasmic enzymes to generate folate intermediates [74]. This however is not the case for all cancer types. For example in kidney cancer cells and cell lines with mutations in cytosolic folate pathway (e.g., PaTu-8988T pancreatic cancer cells) cytoplasmic enzymes contribute equally to NADPH pools instead of using it for anabolism as seen in colon cancer cells [74]. Among the two SHMTs, the expression of SHMT2 has been shown to increase in certain cancers and is regulated by HIF-1α as well as Myc [81]. SHMT2 is an important contributor to mitochondrial NADPH^75^ and is particularly necessary for the maintenance of mitochondrial redox balance in hypoxic tumors [77]. 

*Isocitrate Dehydrogenase (IDH)*: The family of isocitrate dehydrogenases contains three members. Cytosolic IDH1 and mitochondrial IDH2 catalyze NADP+/NADPH-mediated conversion of isocitrate and α-KG [82], whereas mitochondrial IDH3 differs only in using the cofactor NAD+/NADH instead. Point mutations in IDH1, mainly at R132, are observed in a variety of cancers including low-grade gliomas, acute myeloid leukemia, myelodysplastic syndrome, and chronic myelomonocytic leukemias. IDH1 is also highly up-regulated in GBMs, inhibition of which diminishes NADPH, deoxynucleotide and glutathione pools and increases the sensitivity of GBM cells to radiation-damage [83]. In the same cancer type, small molecule GSK864-mediated inhibition of IDH1 favors less differentiated tumor formation and promotes progression of GBM. IDH inhibition also reduces lipid biosynthesis with a reduction in α-KG levels, which alters histone methylation and gene expression in these tumors [84]. In lung cancer cells detached from extracellular matrix, mitochondrial ROS is mitigated by the activity of IDH1 [85]. Jiang et al. identified that IDH1-mediated production of citrate/isocitrate acts as substrate for mitochondrial IDH2 and fuels NADPH production in the mitochondria [85]. Deletion of IDH1, IDH2, or mitochondrial citrate transporter protein (CTP) leads to diminished mitochondrial NADPH levels, increased mitochondrial ROS, and reduced anchorage-independent growth [85]. 

The IDH isoenzymes participate in other interesting biological processes that are not immediately related to the regulation of redox balance. The mutant isoforms exhibit neomorphic activity and convert α-KG and NADPH into the oncometabolite D-2-hydroxyglutarate (D-2-HG) and NADP+ (Figure 4). Conflicting reports exist about the impact of these metabolic alterations on the NADP/NADPH pool [86]. Recently, a new IDH1 inhibitor, AG-120 (Ivosidenib) has been reported which selectively targets multiple IDH1 mutants (R132H, R132C, R132G, and R132L) without side effects on other dehydrogenases [87]. Multiple clinical trials are underway using this inhibitor in IDH1 mutant cancers (Table 1). For a deeper understanding of the ROS unrelated roles of IDH isoenzymes in reductive carboxylation and generation of D-2-HG, the readers are referred to recent excellent reviews on the topic [88,89,90,91,92].

*Nicotinamide Nucleotide Transhydrogenase*: The reducing equivalent stored in NADH can be transferred to mitochondrial NADP+ by using the inner mitochondrial membrane-localized enzyme Nicotinamide Nucleotide Transhydrogenase (NNT) and the proton gradient generated by the ETC (Figure 4). Consistent with its role in antioxidant response, germline deletion of NNT in C57BL/6J mice leads to systemic redox disruption and tissue damage [93,94]. In cancer cells, NNT-mediated generation of NADPH is necessary for glutamine metabolism and reductive carboxylation. Knocking down NNT reduced cell proliferation and glutamine mediated reductive carboxylation, while increasing cellular dependence on glucose utilization in the TCA cycle [95]. Inhibition of reductive carboxylation causes reduced NADPH/NADP+ ratios, which is marginally compensated by a mild increase in the ox-PPP [95]. In mitochondria-defective renal cell carcinoma, increase in reductive carboxylation is accompanied by concurrent oxidative metabolism of α-KG. Mullen et al. reported that in these cancers oxidative metabolism of α-KG produces NADH, which, in the presence of NNT, is converted to NADPH necessary for reductive metabolism [96]. Clearly, in certain cancers, NNT-mediated generation of NADPH is crucial for replenishment of TCA cycle metabolites by glutamine.

Like NNT, NAD Kinase mediates the conversion of NAD^+^ to NADP+. Using High-throughput Mutagenesis and Molecular Barcoding (HiTMMoB), Tsang et al. recently identified mutant NADK^190F^, which exhibits gain of function activity. Although this mutation was present in only one tumor, further analysis in other pancreatic ductal adenocarcinoma (PDA) tumors indicated an increased expression of wild-type NADK [97]. NADK deletion in PDA cells led to increased ROS, inhibition of cell proliferation and tumor volume. Recently, phosphorylation of three N-terminal serine residues of NADK by PI3K-Akt signaling has been shown to activate this enzyme in HEK293T cells [98]. Whether this effect is seen in the case of human cancers, still remains to be answered. 

Maintenance of NADPH homeostasis is another way to support tumor growth especially under stressful growth conditions. Glucose deprivation and matrix detachment causes a reduction in PPP-mediated NADPH production. In this scenario, AMPK phosphorylates acetyl coenzyme-A carboxylases ACC1 and ACC2, reducing NADPH utilization in fatty acid synthesis [99]. Impaired ACC1/2 activity reduces cellular malonyl-coenzyme-A levels releasing the inhibition of carnitine palmitoyltransferase 1 (CPT1), a rate-limiting enzyme for fatty acid oxidation, another route for cellular NADPH synthesis [99]. 

### 3.2. Glutathione

Glutathione (GSH reduced, GSSG oxidized) is a principal cellular antioxidant molecule, and it is critical for detoxification of xenobiotics and maintenance of thiol status of proteins. GSH biosynthesis is initiated when glutamate combines with cysteine in an ATP-dependent reaction catalyzed by glutamate-cysteine ligase (GCL). The product, γ-glutamylcysteine undergoes another ATP-dependent reaction adding glycine to generate GSH. Glutamate can be transported (through EAAT1, SLC1A3, or VGLUT) or synthesized inside the cells by one or other cellular transaminases, depending on the metabolic requirement of the cells. Whereas quiescent mammary epithelial cells, which depend on glutamate dehydrogenase (GLUD) activity to make α-KG, highly proliferative mammary epithelial cells including breast cancer cells prefer transaminase-mediated generation of α-KG [100].

In cancer cells grown in culture, most of the cellular glutamate is generated by glutamine deamidation. Inhibition of glutaminase 1 (GLS1) significantly inhibits the growth of cancer cells [101] at least in part due to the deregulation of GSH biosynthesis [102]. However, in vivo, GLS1 inhibition by CB-839 in genetically engineered mouse models of pancreatic cancer failed to show any therapeutic effect [103]. By studying metabolic adaptations in GLS1-inhibitor-resistant cells, Biancur et al. reported that long-term GLS1 inhibition replenishes cellular Glu, α-KG and succinate pools by Gln-independent and dependent mechanisms [103]. In contrast, lung cancers exhibit increased dependency on GLS1-mediated glutamate generation and these tumors are sensitive to GLS1 inhibition [104,105]. Sayin and LeBoeuf et al. demonstrated that inhibiting GLS1 in KEAP1 mutant lung cancer cells reduces cellular glutamate pools which diminishes its exchange with extracellular cysteine required for GSH biosynthesis [105]. Clearly, the efficacy of targeting glutamine deamidation by GLS1 depends on tumor type and oncogene signaling. Like glutamate, increased uptake and/or synthesis of glycine contributes to enhanced survival of rapidly proliferating cancer cells, with redox maintenance-dependent and independent roles [73,76,106].

Some types of cancer also have an increased dependence on cysteine, the rate limiting amino acid in GSH biosynthesis. For example, gastrointestinal cancer stem cells (CSC) depend on the glutamate-cystine antiporter (system xC^−^, gene *SLC7A11*, Figure 3)-mediated uptake of cystine (the oxidized form of cysteine) and subsequent GSH biosynthesis [107]. In one study, Ishimoto et al. observed that the CSC marker CD44 stabilized xCT, the light chain protein subunit of system xC^−^, to increase cystine uptake [107]. In a subset of triple negative breast cancers, an increased dependence on glutamine uptake (and thus glutamate) was necessary only to acquire environmental cystine to facilitate GSH biosynthesis [108]. Cancer cells expressing low xCT, can derive cysteine from stroma. In chronic lymphocytic leukemia (CLL), cells have low xCT expression and depend on bone marrow stromal cell (BMSC)-generated cysteine for GSH biosynthesis and management of ROS levels [7,109]. 

Several oncogenes have been reported to enhance GSH biosynthesis to favor survival. It has been shown that AKT2-mediated activation of NRF2 signaling increases GSH levels, which combats cellular ROS and aids in 3D and anchorage-independent growth in breast cancer [110]. In pancreatic cancer, oncogenic Kras has been shown to promote NRF2 transcription, which plays an integral role in pancreatic intraepithelial neoplasia (PanIN) formation and progression [46]. 

Glutathione peroxidases (GPX) and glutathione reductases (GSR) are enzymes mediating glutathione recycling in cells (Figure 1). Recent studies have established that increase labile iron pools cause cell death by a unique iron-dependent cell death pathway, ferroptosis [111]. This pathway is characterized by increased oxidation of polyunsaturated fatty acid-containing phospholipids, which are essential for maintaining the architecture of cell membranes [112,113]. To survive in these conditions, cancer cells upregulate lipid peroxidation repair the pathway through increased recycling of glutathione by GPX4. Increase in GPX4 levels promotes cell survival and resistance to ferroptosis in drug-tolerant cancer cells [114,115]. Other cellular GPXs including GPX1-8 also have a well-established role in regulating redox and promoting tumorigenesis in various cancers [116]. GSRs have been linked with reduced ROS levels and resistance to chemotherapy (temozolomide) in GBM [117]. 

### 3.3. Other Antioxidant Enzyme Systems 

The first line of cellular defense against ROS include GPX, peroxiredoxins (PRDXs), and enzymes with high catalytic activity, such as catalases and superoxide dismutases (SODs) (Figure 1). PRDXs are a highly conserved family of thiol-containing peroxidases that catalyze the conversion of H_2_O_2_ to water. PRDXs are maintained in their reduced state by Thioredoxin (TXN1), a group of proteins that mediate reduction of cysteine residues in multiple transcription factors and metabolic enzymes (Figure 1). Oxidized TXNs are reduced by the flavin and NADPH-dependent thioredoxin reductases (TXNRD1). Both TXNs and PRDXs are upregulated in various cancer types [118], and TXN1 and TXNRD1 are regulated by NRF2 [119]. The upregulation of mitochondrial PRDX leads to increased TCA cycle activity and diminished ROS levels [119].

SODs are metalloenzymes present in the mitochondria (Mn SOD, SOD2), cytosol (Cu-Zn SOD or SOD1) or extracellularly (Ec SOD or SOD3). Based on their location, these enzymes mediate cellular signaling by regulating O_2_**^•−^** levels and producing H_2_O_2_ in specific cellular compartments. Previous studies have shown Mn SOD is increased in brain [120], thyroid [121], colorectal [122], and lung cancers [123], and it is activated in breast cancers [124]. In leukemia cells, SOD inhibitor 2-methoxyoestradiol increased O_2_**^•−^** production, autophagy, and cell death when used alone as a single agent [125]. Similar results were seen in HeLa cells treated with SOD inhibitor in combination with ETC inhibitors [126]. Contrary to these findings, increasing Mn SOD levels has been reported to diminish growth in pancreatic cancers [127,128]. Hence, although promising, inhibition of SOD might not be effective therapeutic strategy in all cancers. Like the SODs, catalases are also frequently up regulated in cancer causing resistance to established therapies [129] and mediating ROS detoxification by disproportioning H_2_O_2_ to water (Figure 1). For a detailed understanding of these enzymes, readers are encouraged to see comprehensive reviews on the topic [130,131,132].

## 4. Therapeutic Opportunities Targeting ROS Metabolism

Historically, two general strategies have been applied to target ROS in cancer. The basis for the first strategy was to target the beneficial side of ROS by using antioxidants to reduce ROS levels. However, a growing body of scientific data has demonstrated that this approach may in fact promote tumorigenesis and cancer growth. Thus, as our understanding of ROS biology has matured, the focus of redox targeted therapies in cancer now rely on the opposing strategy to induce ROS directly or to do so indirectly by blocking ROS detoxification, to push cancer cells “over the edge” into cell death.

### 4.1. Inhibiting ROS

In limited quantities and in localized pools, ROS mediates cellular signaling that favors cancer growth [1,133]. Accordingly, it has been proposed that a method to inhibit ROS in cancer cells could impair its growth-promoting effects by blocking ROS-activated signaling pathways. However, due to an increased generalized antioxidant response in tumors, supplementation with antioxidants has proven to be counterintuitive and outright detrimental as a therapeutic approach in cancer [134]. Historically, preclinical studies with antioxidant vitamin E (α-tocopherol) reduced prostate cancer incidence in male smokers [135]. However, the selenium and vitamin E cancer prevention trial (SELECT) demonstrated that individuals on α-tocopherol supplementation had a higher risk of prostate cancer [136]. Similarly, in α -Tocopherol, β-carotene Cancer Prevention Study (ATBC) and β-carotene and Retinol Efficacy trial (CARET), dietary supplementation with β-carotene, or α-tocopherol promoted occurrence of lung cancer in heavy smokers [137,138]. Whilst α-tocopherol scavenges lipid ROS by scavenging ROO**^•^**, β-carotene scavenges both ^1^O_2_ and ROO**^•^** (REF [139]). Like α-tocopherol, vitamin C is a ROS scavenger [140]. Initial clinical studies observed no benefits of dietary supplementation of ascorbate or vitamin C in cancer. However, it was later proposed that this might in part be attributed to the route of administration, as intravenous administration results in a substantially greater therapeutic dose [141]. 

Recently, multiple preclinical studies have reported that antioxidants increase cancer growth and metastasis in cancers [142,143]. For example, Sayin et al. reported that dietary supplementation with antioxidant N-acetyl cysteine (NAC) or vitamin E increases tumor progression in oncogenic KRAS and BRAF-induced lung cancers [144]. In a mouse model of melanoma treatment with NAC or a vitamin E analog, Trolox, had no effect on the growth of primary melanoma tumors but significantly enhanced lymph node metastases [143]. Like NAC, increased NRF2-mediated antioxidant response also increases metastasis in a mouse model of melanoma [145].

It is important to note that the dietary antioxidants utilized in the studies described above (i.e., NAC and vitamin E) inhibit the generalized and growth inhibitory effects of ROS, where localized pools of ROS such as those in mitochondrial remain inaccessible [134]. In a separate study, Liou et al. demonstrated that increased mitochondrial ROS mediates activation of protein kinase D1 and NFκB causing increased EGFR signaling and dedifferentiation of pancreatic acinar cells [146]. The authors observed that treatment with a mitochondria-targeted antioxidant, MitoQ, diminished acinar to ductal metaplasia and hence pancreatic tumor progression [146]. Therefore, for this strategy to be clinically viable, antioxidants that target localized ROS signaling will need to be identified and validated.

### 4.2. Activating ROS

It is well appreciated that most chemotherapies and radiotherapies induce ROS in cancer cells [147,148]. In fact, cancer cells that are resistant to these therapies have often developed mechanisms that inhibit ROS [149]. Based on this, modern studies have aimed to identify molecular mechanisms that facilitate the selective induction of ROS in malignant cells by taking advantage of intrinsic dependences, like the presence and addiction to oncogenes and oncogene-driven pathways. For example, it has been shown that the pro-oxidative properties of vitamin C (ascorbate in the reduced form) selectively kill cancer cells with KRAS pathway mutations in a ROS-dependent manner. Vitamin C is in equilibrium with its oxidized form dehydroascorbic acid (DHA). While classically considered an antioxidant and ROS scavenger [140], when converted to DHA, vitamin C exhibits significant pro-oxidant activity. DHA, and to a much lesser degree ascorbic acid, can enter cells by way of the glucose transporter 1 (GLUT1). Expression of GLUT1 is regulated by several oncogenic signaling pathways, including oncogenic KRAS [150] and is increased in cancer cells. Based on this premise, Yun et al. observed that colorectal cancers with oncogenic KRAS or BRAF mutations have higher sensitivity to vitamin C [150]. GLUT1 expressing cells take up large amounts of DHA. Once inside the cells, DHA consumes NADPH and cellular GSH reservoirs to generate ascorbate. Mouse models of Kras and Braf mutant tumors showed significant response to vitamin C treatment unlike colon cancer cells wild type for the KRAS pathway, which remained less sensitive to this treatment. The genotype-specificity of this treatment could be utilized in the clinical setting to match patients and treatment, a concept that is now being explored in a phase II clinical trial in solid tumors (NCT03146962, Table 1). In a more recent study, Aguilera et al. report that vitamin C causes detachment of oncogenic KRAS from the plasma membrane, resulting in inhibition of MEK-ERK signaling and decreased phosphorylation of pyruvate kinase M2 (PKM2) in colon cancer cells. Reduced phosphorylation of PKM2 diminishes its interaction with β-catenin and TCF/LEF causing a reduction in Myc expression. The authors reported that this reduction in Myc expression, reduced GLUT1 and PKM2-Polypyrimidine tract binding protein 1 (PTB1) expression inhibiting tumor growth [151]. While this study provides correlative evidence that GLUT1 expression and oncogenic KRAS signaling are reduced following treatment with vitamin C, the contribution of GSH depletion and redox imbalance in altered cellular signaling was not overruled. Further, the mechanism by which vitamin C mediates detachment of KRAS from the plasma membrane remains unknown.

In contrast to the above findings, Chen and colleagues reported that extracellular and not intracellular ascorbate selectively kills tumor cells while maintaining the antioxidant effect on surrounding normal tissue [152]. These authors suggest that autoxidation of intravenously administered ascorbate at physiological pH produces H_2_O_2_, which causes apoptosis and necrosis in cancer cells. Further, studies propose that presence of catalytic metals, mainly labile Fe^2+^, accentuates the autoxidation of ascorbate [153]. Tumors have an increase in labile iron pools that could explain the autoxidation of ascorbate to generate H_2_O_2_ and further generation of **^•^**OH via the Fenton’s reaction, selectively killing cancer cells [13]. Unlike the former studies which show increased effectiveness of ascorbate due to increased GLUT1 expression in tumors, these studies propose differential targeting of cancer cells based on their increased ability to generate O_2_**^•−^** and eventually H_2_O_2_ [154]. Clinical trials based on these studies are also underway for pancreatic and lung cancers to study the effectiveness of ascorbate in combination with chemotherapy (Table 1).

Another strategy to induce ROS specifically in cancer cells takes advantage of a gene that is over-expressed in many cancers, NADP quinone oxidases 1 (NQO1). The Kras signaling pathway downstream of NRF2 leads to NQO1 overexpression [155,156,157]. β-lapachone (β-Lap or ARQ 501) is a prodrug that is metabolized by NQO1 in a futile cycle that consumes reducing potential in the form of NADH or NADPH, and the associated electrons are transferred to oxygen to generate O_2_**^•−^**. This then completes the cycle ultimately regenerating β-Lap [155,158,159]. This simultaneous burst of ROS generation and reducing potential consumption leads to tumor selective, ROS-mediated cell death [160]. This process is mediated by and requires NQO1 expression, and β-Lap is now being tested in clinical trials in NQO1-positive tumors (Table 1). Despite the promise of this agent, it has a narrow therapeutic window and causes dose-limiting methemoglobinemia. To address this, Chakrabarti et al. tested β-Lap in combination with an inhibitor of GLS1 in pancreatic cancer. The authors found that combining β-lap with the GLS1 inhibitors CB-839 or bis-2 (5-phenylacetamido-1,2,4,-thiadiazol-2yl) ethyl sulfide (BPTES) depleted cellular NADPH pools and yielded promising results in preclinical studies in pancreatic cancer [102]. Of note, the dose of β-Lap utilized in this study was decreased substantially and suggests that such a rationally designed approach may maximize the utility of β-Lap, while minimizing dose limiting side effects. In a similar study using CB-839 alone or in combination with paclitaxel, significant antitumor activity in mice bearing triple negative breast tumors with high expression and activity of GLS splice variants were observed [161].

A parallel strategy to increasing ROS in cancer cells is to inhibit the pathways that manage redox burden. NRF2 regulates the transcription of multiple genes mediating the cellular antioxidant response, including enzymes in the serine glycine biosynthesis pathway. Targeting the transcription ability of NRF2 can cripple the antioxidant machinery, causing cell death. Bollong et al. utilized a high throughput small molecular screen to identify small molecule inhibitor of NRF2 activity [162]. They reported that treatment with AEM1, an inhibitor of NRF2 transcription activity, reduced anchorage-independent growth and increased sensitivity to chemotherapy in A549 cells [162]. Similarly, Bar-Peled et al., by applying cysteine proteomics, identified the nuclear receptor NROB1 as a key component of NRF2 transcription regulator complex that promotes anchorage independent growth in KEAP1 mutant NSCLC [163]. Loss of liver kinase B1 (LKB1, also known as serine/threonine kinase 11 or STK11) in non-small cell lung cancer (NSCLC) has been shown to enrich these tumors with KEAP1 mutations, making these cancers vulnerable to inhibition of NRF2-mediated antioxidant response [164]. Since KEAP1 mutant NSCLC are dependent on glutamine to generate cellular GSH [105], loss of LKB1 increases the sensitivity of NSCLC to glutaminase inhibitors. Targeting KEAP1-inhibiting proteins, especially those with the least physiological implications, can also mitigate NRF2 activity. Since therapeutic targeting of NRF2 is not practical regulating KEAP1 has the potential to inhibit cancer-specific NRF2 signaling without affecting normal cells.

## 5. Concluding Remarks and Future Directions

ROS generation, signaling, and regulation are highly orchestrated physiological events that are modified in cancer to favor its survival, growth, and progression. There are several aspects of cancer biology where a more detailed understanding of the role of redox balance is likely to provide new targets to inhibit cancer, avoid therapeutic resistance, and achieve clinically beneficial results. Namely, the dependence on antioxidant pathways can vary based on tumor type and stage. For example, targeting GSH biosynthesis provided beneficial results only before the onset of breast tumors in mice; in established tumors, redundancy of GSH and TXN provide an inherent mechanism of resistance [57]. Another significant aspect to consider is inter and intra-tumoral genetic and metabolic heterogeneity in cancers [165]. Targeting redox dependencies in one subset of cancer cells could spare or lead to development of resistance in other subpopulations. In fact, as exemplified in pancreatic cancers, tumor initiating and stem-like cells have increased dependence on mitochondrial respiration, unlike bulk tumor population, which rapidly proliferates and depends on glycolysis [166,167]. There are also distinctions in redox regulation in cells that lie within the tumor relative to those in circulation and bound for metastatic colonization [143,144,145]. Hence, a composite understanding of ROS axis will help identify the opportunities and challenges in developing more clinical effective ROS-based strategies.

## Figures and Tables

**Table 1:** Ongoing clinical trials with therapies directly or indirectly modulating ROS metabolism.

Molecule	Mechanism	Phase	Combination (Cancer type)	Clinical Trial ID
**Inhibiting ROS:**				
Vitamin E, Tocotrienol	Lipid Antioxidant	Phase 2	Stereotactic Ablative Radiotherapy and Pentoxifylline (Lung cancer)	NCT01871454
Vitamin A (high dose)	Lipid Antioxidant	Early Phase 1	Neoadjuvant (Lung cancer)	NCT03870529
N-acetyl cysteine	General antioxidant, to alleviate the side effects of standard therapy	Phase 1,2	Low dose phase 1, high dose with paclitaxel Phase 2 (solid tumors)	NCT03492047
		Phase 2	Chemotherapy and radiation (head and neck)	NCT03982537
**Activating ROS:**				
AG-120 (Ivosidenib) or AG881	Suppression of D-2-HG in IDH1 mutant cancers	Phase 1	(Low grade glioma)	NCT03343197
		Phase 1	(myeloid cancers)	NCT03564821
		Phase 1	(AML)	NCT02074839
		Phase 1	(solid tumors)	NCT02073994
		Phase 1,2	Azacitidine, Venetoclax (AML)	NCT03471260
		Phase 3	Azacitidine (AML)	NCT03173248
ARQ761 (β-lapachone)	Nqo1 Substrate, causes oxidoreduction and production of O_2_^•–^, depletion of NAD(P)H	Phase 1	(solid tumors)	NCT01502800
		Phase 1	PARP inhibitor Olaparib (solid tumors)	NCT03575078
		Phase 1	Gemcitabine, nab-paclitaxel (PDA)	NCT02514031
Ascorbate (High dose ascobic acid)	NADPH and GSH depletion, Increasing H_2_O_2_ Levels	Phase 1,2	(soft tissue sarcomas)	NCT03508726
		Phase 1	Gemcitabine and radiation (PDA)	NCT01852890
		Phase 2	nab-paclitaxel with gemcitabine (PDA)	NCT02905578
		Phase 2	Gemcitabine and radiation (PDA)	NCT03541486 (XACT-PANC-2)
		Phase 1	Temozolomide and radiation (GBM)	NCT01752491
		Phase 2	Temozolomide and radiation (GBM)	NCT02344355
		Phase 1	low dose melphalan + high dose ascorbate acid (Myeloma)	NCT03602235
		Phase 1,2	Tyrosine Kinase Inhibitors (Lung cancer)	NCT03799094
		Phase 2	Radiotherapy with carboplatin and paclitaxel (Lung cancer)	NCT02905591 (XACT-LUNG)
		Phase 2	carboplatin and paclitaxel (Lung cancer)	NCT02420314
		Phase 2	(solid tumors)	NCT03146962
CB-839	Glutaminase Inhibitor	Phase 1,2	Nivolumab (RCC, Lung)	NCT02771626
		Phase 2	Everolimus (RCC)	NCT03163667
		Phase 2	Cabozantinib (RCC)	NCT03428217
		Phase 1,2	Osimertinib (Lung cancer, EGFR mutation)	NCT03831932
		Phase 1	Niraparib (platinum resistant BRCA wild-type ovarian cancer)	NCT03944902
		Phase 2	paclitaxel (TNBC)	NCT03057600
		Phase 1,2	Panitumumab and irinotecan hydrochloride (CC)	NCT03263429
		Phase 1,2	Capecitabine (solid tumors, CC)	NCT02861300
		Phase 1	Carfilzomib, and dexamethasone (plasma cell myeloma)	NCT03798678
		Phase 1	Single agent, combination with standard chemotherapy (Solid tumors)	NCT02071862
		Phase 1,2	CDK4/6 Inhibitor Palbociclib (solid tumors)	NCT03965845
		Phase 1,2	Talazoparib (solid tumors)	NCT03875313
		Phase 2	(KEAP1, NRF2, STK11/LKB1 mutant solid tumors)	NCT03872427

AML= acute myeloid leukemia, GBM = glioblastoma multiforme, MM = multiple myeloma, PDA = pancreatic ductal adenocarcinoma, RCC = renal cell carcinoma, TNBC = triple negative breast cancer, KEAP1 = Kelch-like ECH-associated protein 1, NRF2 = Nuclear factor erythroid 2-related factor 2, STK11/LKB1 = Serine/Threonine Kinase 11/Liver Kinase B1.
